# High Prevalence of Vitamin D Deficiency in Cambodian Women: A Common Deficiency in a Sunny Country

**DOI:** 10.3390/nu8050290

**Published:** 2016-05-12

**Authors:** Geoffry Smith, Sunil J. Wimalawansa, Arnaud Laillou, Prak Sophonneary, Samoeurn Un, Rathavuth Hong, Etienne Poirot, Khov Kuong, Chhoun Chamnan, Francisco N. De los Reyes, Frank T. Wieringa

**Affiliations:** 1 International Life Sciences Institute, South East Asia Region, 9 Mohamed Sultan Road #02-01, Singapore 238959, Singapore; geoffsmith@ilsisea.org.sg; 2 Essential Micronutrients Foundation, 3 Pickering Street, #02-36 Nankin Row, China Square Central, Singapore 048660, Singapore; 3 Cardio Metabolic Institute, Medicine, Endocrinology & Nutrition, 661 Darmody Avenue, North Brunswick, NJ 08902, USA; suniljw@hotmail.com; 4 United Nations Children’s Fund (UNICEF), Maternal, Newborn and Child Health and Nutrition Section, No. 11 street 75, Phnom Penh 12202, Cambodia; sun@unicef.org (S.U.); epoirot@unicef.org (E.P.); 5 National Nutrition Program, Maternal and Child Health Center, No. 31A, Rue de France (St. 47), Phnom Penh 12202, Cambodia; sophonprak@gmail.com; 6 ICF International, 530 Gaither Road, Suite 500, Rockville, MD 20850, USA; rathavuth.hong@icfi.com; 7 Department of Fisheries Post-harvest Technologies and Quality control (DFPTQ), Fisheries Administration, Ministry of Agriculture, Forestry and Fisheries (MAFF), 186 Preah Norodom Boulevard, Phnom Penh 12000, Cambodia; kuong.kh@gmail.com (K.K.); chhounchamnan@gmail.com (C.C.); 8 Nutrition, Exercise and Sports, University of Copenhagen, Bülowsvej 17, Frederiksberg C DK-1870, Denmark; 9 University of the Philippines, Diliman, Quezon City 1101, Philippines; fndelosreyes@up.edu.ph; 10 Institute of Research for Development (IRD), UMR Nutripass IRD-UM2-UM1, Montpellier 3400, France; franck.wieringa@ird.fr

**Keywords:** vitamin D, national survey, Cambodia, Demographic Health Survey Nutrition, South East Asia

## Abstract

Recent studies have shown that in spite of being generally close to the equator; vitamin D deficiency is common in South East Asian countries. In order to quantify micronutrient status for women and children in Cambodia; a nationally-representative survey was conducted in 2014 linked to the Cambodian Demographic Health Survey. The countrywide median of 25(OH)D was, respectively, 64.9 and 91.1 nmol/L for mothers and children. Based on The Endocrine Society cutoffs (>50<75 nmol/L = insufficiency; ≤50 nmol/L = deficiency); 64.6% of mothers and 34.8% of their children had plasma vitamin D concentrations indicating insufficiency or deficiency. For deficiency alone, 29% of the mothers were found to be vitamin D deficient, but only 13.4% of children. Children who live in urban areas had a 43% higher rate of vitamin D insufficiency *versus* those who live in rural areas (OR; 1.434; 95% CI: 1.007; 2.041). However, such differences were not observed in their mothers. The high prevalence of vitamin D deficiency is likely in part due to lifestyle choices, including sun avoidance, increasingly predominant indoor work, and covered transport. These survey findings support the need for a broader national Cambodian study incorporating testing of adult men, adolescents and the elderly, and encompassing other parameters such as skeletal health. However, the data presented in this study already show significant deficiencies which need to be addressed and we discuss the benefit of establishing nationally-mandated food fortification programs to enhance the intake of vitamin D.

## 1. Introduction

Vitamin D status has only relatively recently received attention in South East Asia [1]. The region spans the equator to roughly 28° north and 10° south latitude, so sunlight at a low zenith angle is common. Vitamin D in humans is synthesized in the epidermis from 7-dehydrocholesterol (7-DHC) catalyzed by sun exposure containing UVB photons (290–315 nm). It has been assumed that sufficient sunlight in the South East Asian region would insure adequate vitamin D levels for most people. The typical rating of skin pigmentation of South East Asian populations is IV on the Fitzpatrick scale (moderate brown skin) [2], requiring more sunshine exposure than Caucasian populations to generate adequate vitamin D, but not long exposure. At the latitude of Cambodia (roughly 11.5°–13° North), it is estimated that 15–30 min exposure in the morning or late afternoon, or 10–20 min at midday of 25% of the skin surface a few times a week will ensure adequate vitamin D levels [3].

However, recent studies in South East Asian countries, including nationally-representative surveys among women and children, in particular, have shown a higher than expected prevalence of vitamin D deficiency in the region [4,5,6,7,8]. With a cutoff of <50 nmol/L (20 ng/mL), these surveys have shown that vitamin D deficiency is widespread in the four countries studied. For example, 44.2% in Indonesian children 6 months–12 years old were vitamin D deficient [7] (including 66.9% of rural girls aged 7–12) and 47.5% in Malaysian children [8] (including 66.7% of urban girls aged 7–12). These nationally-representative results confirm other smaller clinical and cross-sectional studies conducted in the region, which demonstrated a high prevalence of vitamin D deficiency [9,10,11,12,13].

Some public health recommendations for vitamin D are based primarily on requirements for bone health. However, vitamin D deficiency is often associated in epidemiological studies with higher all-cause mortality, as well as an increased risk of many types of cancer, including breast, colorectal, cervical, ovarian, pancreatic, and others [14,15]. These associations for colorectal cancer have also been found in Asia in case-controlled studies and meta-analyses [16,17,18]. In addition to cancer, observational studies have found that a higher vitamin D status is associated with a decreased risk of mental disorders, infectious disease, cardiovascular disease, type 2 diabetes mellitus, and autoimmune disorders [19,20,21]. Randomized clinical controlled studies have not always confirmed these associations (perhaps in some cases also due to latency issues) but some Randomized Controlled Trials have shown an improvement in immune response with higher levels of vitamin D [22,23].

### Vitamin D Deficiency and Skeletal Health

Historically, the predominant observed consequence of having vitamin D deficiency was development of osteomalacia in adults and rickets in children [24]. No national monitoring system exists in Cambodia to know the prevalence of those issues; however, several reports from pediatricians have described the existence of rickets in children. Adolescence is a key period for bone development, as roughly half of adult peak bone mass (PBM) is formed during this phase of life. Additionally, the time of the fastest bone mass accumulation rate is generally in the years around puberty, when up to 25% of PBM is formed [25]. Lifetime PBM is generally achieved in the early 20s in most individuals (however, one study in Vietnam has shown a later PBM age of 27–29 years [26]). Bone mass generally starts to decline after the fifth decade of life [27].

Osteoporosis, a disease of reduced bone density and strength, is increasingly common in South East Asia, and is a major contributor to fatal outcomes from falls [28]. Aging is a major risk factor for osteoporosis; in general, populations in the region are getting older on average, although the median age in Cambodia is low (about 25 years). Development of osteoporosis is greatly influenced by physical activity, vitamin D status, and calcium intake, but the likelihood of developing the disease is also dependent on bone development in adolescence and maintenance in middle life [25,29].

The key detrimental outcome of osteoporosis is the risk of fractures, especially hip fractures which result in high morbidity and mortality. An estimated 20% of women who sustain a hip fracture survive less than 12 months and an additional 20% or more require long term care [28]. The best diagnostic tool for osteoporosis is the use of dual energy X-ray bone absorptiometry or DXA, according to the World Health Organization’s report on the “Prevention and Management of Osteoporosis” [30]. There is only one identified DXA machine available in Cambodia (0.06 per 1.0 million people), which is insufficient to give a good estimation of the prevalence of osteoporosis for the country.

In view of the studies in the region finding higher than expected vitamin D deficiency, recommendations were made to conduct nationally-representative surveys of vitamin D status in all South East Asian countries. Consequently, a nationally representative survey was carried out in 2014 in Cambodia for a number of micronutrients, including vitamin D in women and children. This paper reports the vitamin D data from that survey.

## 2. Experimental Section

### 2.1. Study Design and Sampling

In 2014, The Cambodian Demographic Health Survey (CDHS) conducted a nationally-representative survey of women and men between the ages of 15 and 49 years of age, in 16,356 households [31]. The sample participants were selected in two stages. In the first stage, 611 villages (also known as clusters or enumeration areas) were selected with probability proportional to village size. Village size is the number of households residing in the village. Then, a complete mapping and listing of all households existing in the selected villages was conducted. The resulting lists of households served as the sampling frame for the second stage of sample selection. Households were systematically selected from those lists for participation in the survey.

The 2014 CDHS included a micronutrient component which was implemented in one-sixth of the clusters selected for the main survey, collecting data only in women from the survey and adding children under five years of age. In these clusters, urine and blood samples were collected from women who had given birth in the five years preceding the survey and from their children aged 6–59 months. Only clusters where data on anemia was collected were eligible for inclusion in the micronutrient survey.

The sample size for this study was calculated to obtain a margin of error of 3% at the national level and 5% at urban and rural level. Anticipating up to 20% refusal/dropouts, 935 mothers and 1096 children were recruited. The number of blood samples that could not be collected from women was 210 and from children 315 samples, resulting in a net number of samples from women of 725 and 781 from children. The women of reproductive age were selected according to the following criteria: (i) mother of the child 6-59 months surveyed; (ii) from households having participated in CDHS 2014 the previous week(s); (iii) no evidence for severe or chronic illness; and (iv) having agreed to, and signed the informed consent form to participate.

The criteria for children less than five years to participate in the survey were as follows: (i) from households having participated in CDHS 2014 the previous week(s); (ii) aged between 6 and 59 months of age; (iii) no evidence of severe or chronic illness, including congenital abnormalities, mental or severe physical handicap; and (iv) written informed consent signed by at least one parent.

### 2.2. Blood Sampling and Analysis

The micronutrient survey was conducted at each house in each cluster in the morning. Five survey teams were set up for the study. One survey team visited only one cluster. Each survey team consisted of three persons: one team leader (and interviewer) and two nurses for the biological sampling. After checking for the household (HH) code and individual (ID) codes, the questionnaire was given to the selected mother/caretaker of the selected children. Blood samples were drawn by venipuncture in a trace-element free vacutainer with heparin as anticoagulant (Vacuette, Greiner Bio One) by experienced nurses: 6 mL for women of reproductive age (WRA) and 4 mL for children. After the morning phlebotomy, blood samples were then stored in the dark in a cool box and transported to the laboratory of Provincial Health Center (PHC) within 6 h of blood collection.

After arrival of the blood samples in the provincial health center, blood samples were centrifuged (3000 g for 10 min) and plasma (1 tube of 3 mL) was collected and frozen at −20 °C. Upon finalizing one cluster, all samples were sent to the laboratory of DFPTQ (Department of Fisheries Post-Harvest Technologies and Quality Control, Fisheries Administration, a government research, technology, and quality organization). Blood samples were thawed and divided in four tubes at the laboratory of DFPTQ and then transported on ice to the Cambodian Institut Pasteur for the analysis of vitamin D content. Plasma 25-hydroxyvitamin D (25(OH)D) was measured through a solid phase Enzyme Linked Immunosorbent Assay (ELISA) performed on microtiter plates (Roche Diagnostics E170; F. Hoffmann-La Roche AG, Basel, Switzerland).

### 2.3. Statistical Analysis

Data analysis was performed with SPSS software (SPSS, V20; IBM, Chicago, IL USA). We defined vitamin D status as:

- Severely deficient: 25(OH)D ≤ 25 nmol/L
- Deficient: 25(OH)D > 25 nmol/L and ≤50 nmol/L
- Insufficient: 25(OH)D >50 and <75 nmol/L
- Sufficient: 25(OH)D was ≥75 nmol/L

According to the recommended values from the Endocrine Society [32] (and expert assessment for severe deficiency [33], but other values are recommended by other organizations) [34,35,36].

Descriptive statistics were used to examine 25(OH)D relationships. Vitamin D concentration for mothers was not normally distributed according to the test of Kolmogorov-Smirnov (*p* = 0.000) and, therefore, only non-parametric tests were performed. Median and interquartile range values of the vitamin D concentrations are presented and disaggregated by wealth index, and urban/rural status. Vitamin D concentration (continuous variable) was compared by groups by using the non-parametric Mann-Whitney U-test (two groups) or Kruskal-Wallis for independent variables. For children, vitamin D concentrations followed a normal distribution (Kolmogorov-Smirnov test, *p* = 0.669) and, therefore, parametric tests were performed to compare groups (*i.e.*, *t-*test and ANOVA test). Odds analysis was also performed to examine the association between having an insufficient vitamin D status (<75 nmol/L) and several factors. Results were considered significant at *p* < 0.05.

Socio-economic status was calculated using the Demographic Health Statistic (DHS) Wealth Index [37] to divide households surveyed into five socio-economic groups: the “extreme poor” (category 1), the“ poor” (category 2), the “intermediate” (categories 3 and 4), and the “wealthiest” (category 5). The Wealth Index was constructed from recorded data on household assets, such as tables, chairs, refrigerator, air conditioners and beds, and also from housing conditions (materials of house floor, house roof, main wall) and facilities (energy for cooking, electricity and latrines); income and expenditure were not used.

### 2.4. Ethical Issues

The Scientific Committees of the Ministry of Health (Phnom Penh Cambodia) reviewed and approved the study protocol. All women were informed verbally and in writing about the aims and procedures of the study, and written informed consent was obtained from all women and children (via their mother or guardian) before enrollment.

## 3. Results

A. Women—after dropouts/refusals, there were 725 female participants, all in the 15–49 age group (estimated median age 30 years, 25th percentile–75th percentile (_P25–P75_): 25, 34; age data from only 481 mothers), orally stated not to be pregnant. Median weight was 50.3 kg (_P25–P75_: 45–57.2 kg) while median height was 152.7 cm (_P25–P75_: 149.4–156 cm). Median BMI was 21.48 (_P25–P75_: 19.53–24.26). For the women, the countrywide median 25(OH)D was 64.9 nmol/L (_P25–P75_: 47.5 nmol/L–88.2 nmol/L). Overall mean vitamin D concentration was 69.7 nmol/L (SD ± 31.2 nmol/L) (Table 1). Median 25(OH)D was significantly different among women according to their region of living (*p* < 0.001), with a median of 57.1 nmol/L for those living in the northeast (_P25-P75_: 41.9–73.8 nmol/L), but 73.4 nmol/southwest (_P25-P75_: 54.4–99.6 nmol/L). Consequently, vitamin D deficiency is associated with the region where the women live (*p* = 0.001). The highest prevalence was in the north (39.2%) followed by northeast (37.7%) and southeast (29.4%). Below the national prevalence estimate were the west region (22.8%) and the southwest region (21.1%). No significant differences of the median 25(OH)D were observed between socioeconomic groups and living areas (urban *vs.* rural) (*p* = 0.301).

There was also a wide difference of median 25(OH)D concentrations between provinces, with Kampong Cham province having a median of 41.5 nmol/L, while Pailin province was measured with a median of 100 nmol/L, however, there was insufficient statistical power to establish significance at the province level due to the number of provinces. Thus, analysis of differences at the region level was done.

Using the different cut-offs to show vitamin D status, 29% of the mothers were considered to be vitamin D deficient (<50 nmol/L, Table 2), including 4.1% severely deficient (<25 nmol/L), while 64.6% showed vitamin D deficiency or insufficiency with a cutoff of <75 nmol/L. The prevalence of vitamin D deficient women (<50 nmol/L) was not significantly different between mothers living in rural areas compared to urban areas (*p* = 0.301) or for those belonging to the poorest social-economic class compared to the wealthiest group (*p* = 0.358).

B. Children—there were 781 child subjects, primarily aged six months to five years old, but with 128 subjects somewhat over five years old. Median weight was 12.8 kg (_P25-P75_: 10.5–14.6 kg) while median height was 93.6 cm (_P25-P75_: 83.6–100.4 cm). Overall mean vitamin D concentration was well above the sufficiency cutoff (of >75 nmol/L) at 91.1 nmol/L (SD ± 37.3 nmol/L) (Table 3). Median vitamin D concentration was 89.5 nmol/L (_P25-P75_: 64.7–116 nmol/L). Unlike for women, significant differences were observed in the concentration of vitamin D according to urban/rural status. For example, the mean 25(OH)D concentration was significantly lower among children if they were living in an urban area compared a rural area (*p* < 0.001), with a mean of 83.3 ± 31.8 nmol/L for urban populations, and 93.1 ± 38.4 nmol/L for rural populations. Comparative analysis showed that mean 25(OH)D concentration was highest in the youngest children and significantly decreased with age, from 120.2 nmol/L for the youngest children, to only 82.8 nmol/L for the older children (*p* < 0.0001).

In general, the prevalence of vitamin D deficiency for children was much lower than in the women; only 13.4% were deficient (<50 nmol/L) and 2.9% were severely deficient (<25 nmol/L) (Table 4). The prevalence of vitamin D deficient children was not significantly different between children living in rural *versus* urban areas (*p* = 0.234) or for those belonging to the poorest social-economic class compared to the wealthiest group (*p* = 0.076). However, the odds ratio (OR) of vitamin D insufficiency was statistically significant at 1.434 (95% CI: 1.007, 2.041). This indicates that children in urban locales tend to be 43% more likely to be vitamin D insufficient than their rural counterparts.

There were significant differences in the vitamin D concentration across age groups (*p* = 0.001). Highest status was seen among the 6–11 months’ group at 119.8 nmol/L median value, followed by age 12–23 months with median value 104.1 nmol/L, then by 24–59 months at 87.2 nmol/L. The 60+ months age group had relatively lower values at a median of 80.9 nmol/L. Vitamin D deficiency followed this same pattern in inverse (*p* = 0.001), with the lowest prevalence in the youngest group (7.3%), followed by 12–23 months (11.3%), then 24–59 months (12.9%), and the highest prevalence among the 60+ months subjects (19.6%).

As opposed to mothers, the likelihood of having vitamin D insufficiency (<75 nmol/L) was higher for children if they lived in urban areas as compared to rural areas. However, even if the odds ratio (OR) of having vitamin D deficiency is 1.20 for children living in urban areas compared to their rural counterparts, this is not statistically significant (95% CI for OR: 0.742–1.963). We also found that vitamin D deficiency was associated with the region where the children live (*p* = 0.001). The highest prevalence of vitamin D deficiency was in the north (20.1%), followed by the northeast (17.4%) and southeast (16.7%). Below the countrywide prevalence estimate are the southwest (8.4%) and west (4.6%).

This study did not cover adolescents. However, with almost 30% of the women in our study with levels considered deficient (<50 nmol/L) and with no difference by wealth grouping, our study strongly suggests the need to follow adolescents as this is a key age for bone mass accumulation. As observed among the children in this survey, the concentration of 25(OH)D decreased significantly with age. If this is continuing until adolescence in Cambodia, it would be expected to have an impact on bone development, as well as future development of osteoporosis and fractures.

## 4. Discussion

To our knowledge, this survey is the first study to report nationally representative data on vitamin D status in Cambodia. Using the Endocrine Society cutoffs, just under one-third of the women were deficient (29.0%), as well as 13.4% of the children. Almost 2/3 of women (64.9%) and more than a third of their children (34.8%) were vitamin D insufficient or deficient (<75 nmol/L). However, the prevalence of severe vitamin D deficiency among women and children (<25 nmol/L) is below 5% (2.9% for children and 4.1% for mothers).

As there is no formal agreement on the appropriate cutoffs for vitamin D deficiency and insufficiency in Cambodia, or even South East Asia, we used the IOM (Institute of Medicine) recommendation and The Endocrine Society recommendations, as well as three expert reviews for comparison [32,33,34,35,36]. The IOM cutoffs [34] are based on the need for vitamin D in bone heath, including bone formation in childhood and young adulthood, and bone maintenance in middle and later life. The IOM recommendations are not intended for individuals but for public health use by the governments in North America. Thus, it is not a recommendation directly applicable outside of North America. Conversely, The Endocrine Society (TES) Guidelines are for patients, but are not intended for specific vulnerable populations, nor for ethnic groups [32].

A recent study among black and Caucasian populations in the U.S. found that while black Americans, compared with whites, had lower levels of total 25(OH)D and vitamin D receptors (VDR), they, nevertheless, had similar concentrations of estimated bioavailable 25(OH)D [38]. These differences were attributed to racial differences in the prevalence of genetic polymorphisms. Similar studies have yet to be done in South East Asian populations, but a recent study in Thailand did find that vitamin D binding protein gene polymorphisms were associated with vitamin D deficiency, suggesting these polymorphisms are a risk factor for vitamin D deficiency in Thais [39].

Other issues that need attention are calcium intakes and metabolism in South East Asian populations. Since dairy consumption is low in Asia, calcium intakes are often considerably lower than those of European or American consumers. However a U.S. study of Chinese-American boys and girls (11–15 years) showed more efficient calcium metabolism than for their Caucasian counterparts, and that calcium intakes to achieve maximal calcium retention were lower—1100 mg/day in boys and 970 mg/day in girls, *versus* the recommendation for Caucasians of 1140 mg/day for boys and 1300 mg/day for girls [40].

The high percentage of the population with insufficient vitamin D concentration is worrisome and previously unexpected in sunny countries such as Cambodia. According to international studies, the amount of vitamin D generated is determined by the availability of sunlight, the intensity and zenith angle, the time of exposure, as well as the pigmentation of the skin and the percentage of body exposed [2]. Higher levels of skin pigmentation reduce the conversion rate to vitamin D, but the amount of exposure time needed to prevent deficiency is relatively low, especially in the South East Asian latitudes.

An exposure of sunlight on the skin of a minimal erythemal dose (MED—the amount of radiation needed to produce capillary engorgement) would generate vitamin D equivalent to an oral dose of between 10,000 and 25,000 IU [41]. Therefore, the low level of exposure needed to generate adequate vitamin D from sun exposure may explain why children in Cambodia largely have adequate levels. Walking to school and playing outdoors should allow these exposures easily. For women, cultural and lifestyle factors may be decreasing sun exposure. Although farming remains the largest activity in Cambodia, there is increasing urbanization and factory work which would reduce sun exposure.

In those high and low latitude countries with large Caucasian or lower Fitzpatrick scale skin pigmentation, there is concern about added sun exposure contributing to melanoma (skin cancer). The evidence is not completely clear, as many people who get melanoma have little sun exposure, and often melanomas occur on parts of the body that receive little or no sun [42,43]. It is clear that the rates of melanoma are significantly lower in populations with higher skin pigmentation.

A recent survey in Singapore estimates that melanomas were occurring in Singaporeans at 1% of the rate of Caucasians in North America [44]. It is clear that a relatively small increase in the sun exposure of the adult female population in Cambodia could successfully address vitamin D deficiency [3], and two recent commentaries have recommended sunshine as the preferred public health approach [45,46]. However, it may be difficult to change the lifestyle and aesthetic priorities in South East Asia even with better education about the risks of vitamin D deficiency [47].

Another theoretical approach to improving vitamin D status and preventing deficiencies is through the daily diet. The vitamin D intake level recommend by the IOM for North American populations is 600 IU/day for most individuals, increasing to 800 IU/day for those over 70 years of age (due to lower conversion rates [48]. These levels correspond to 15 µg and 20 µg/day. There is no survey of vitamin D intakes in Cambodia, but recent studies in Vietnam have shown intakes for women of 0.15 µg/day, or less than 1% of the recommended intake for North Americans [6].

A number of foods can be good sources of vitamin D. [3,20]. These foods include salmon, mackerel, sardines, canned tuna, sun dried shitake mushrooms, and egg yolks, and some others [33,49]. The levels of vitamin D in these foods can vary greatly. For example, on average, 200 g of wild salmon can provide 600 to 1000 IU, so consuming this amount 4 to 6 times a week would provide most of one’s vitamin D requirement. However, farmed salmon generally has vitamin D levels of 100–250 IU of vitamin D, so the consumption levels needed to maintain the same intake as for wild salmon would be 2.4 to 6 times as much [50]. Similarly, sun-dried shitake mushrooms can have as much as 1600 IU per 200 g, but fresh shitake mushrooms only contain about 100 IU per 200 grams [51]. Unfortunately, none of these naturally vitamin D rich foods are very common in the Cambodian diet, so, at present, simple dietary diversification cannot reliably address the current deficiency levels in Cambodian women.

Given the limited number of foods with high intrinsic levels of vitamin D, in many high and low latitude countries a range of foods are fortified with vitamin D. In principle, these fortified foods can provide increased intakes of vitamin D [52]. The most common of these is milk, followed by other dairy products such as cheese and yoghurt. However, dairy products are not widely consumed in Cambodia at present. Alternative vehicles for food fortification could be edible oil. A recent review has shown for Vietnam and Indonesia that edible oil fortified with vitamin D could provide as much as 50% of the Dietary Reference Intake (DRI) for a high percentage of the population [53].

### Limitations of the Study

The population analyzed for this article is a subsample of the Cambodian Demographic Health Survey (CDHS). The broader survey included men, women and children, but for cost reasons only women and children under five years of age were included in our study on vitamin D. In high northern and southern latitudes, vitamin D status is affected by seasonality due to sun variability, but this effect should be very moderate in Cambodia due to the low latitude and relatively uniform UV Index values.

Finally, our study used an ELISA test method to measure vitamin D status, while the most accurate test method is LC/MS-MS [54]. Validation of HPLC methods have also shown repeatable results. ELISA methods generally have lower analytical sensitivity at the lower end of the measuring range and greater internal variability [55].

## 5. Conclusions

An increasing number of surveys in South East Asia are showing high levels of vitamin D deficiency, perhaps due to lifestyle changes leading to reduced sun exposure—working in offices or factories, rather than in open air agriculture, traveling by car or bus to school for children, *etc.* Although the exact cutoffs for vitamin D deficiency in Cambodia are not yet clearly defined, this present survey of vitamin D status in women and children in Cambodia confirm those trends, similar to most of the other South East Asian countries that have been surveyed so far. Although nationally-representative bone health data is not available for Cambodia, based on numerous studies of vitamin D and bone health, there may already be a need to consider implementation of food fortification or any other strategy to increase the intake of vitamin D.

While there is strong evidence that the presently-reported levels of vitamin D deficiency need to be addressed, a number of issues need further study in Cambodia and South East Asia. Additional studies are needed in South East Asia to clarify the optimal requirements and intakes. As mentioned above, there is no systematic collection of bone health data for Cambodia and this is needed to understand current prevalence and trends of rickets, osteoporosis, and osteomalacia. Although the exact cutoffs for vitamin D deficiency in Cambodia are not yet clearly defined, we conclude that sufficient deficiency exists to consider implementation of food fortification with vitamin D. Finally, in order to monitor trends in Cambodia, surveillance of vitamin D status needs to be broadened to include adult men and adolescents of both sexes, and should be done regularly to monitor trends and the effectiveness of any steps taken to address the deficiencies.

## Figures and Tables

**Table 1:** Comparison of relationships between vitamin D concentration status and living area (urban/rural), wealth grouping, and geographic region, among women.

Comparisons: Women (1)		Vitamin D (1)						*p*-Value ^1^
		*n*	Mean (nmol/L)	SD (nmol/L)	Median (nmol/L)	25th Percentile P_25_ (nmol/L)	75th Percentile P_75_ (nmol/L)	
Total		725	69.7	31.2	64.9	47.5	88.2	-
Living Area	Urban	148	67.7	29.7	60.6	46.6	85.7	0.301
	Rural	577	70.2	31.6	66.8	47.8	89.0	
Wealth Group	Poorest	155	71.7	34.2	66.6	47.8	92.3	0.358
	Poorer	152	72.3	31.4	69.0	50.3	93.5	
	Middle	131	67.7	30.2	63.0	43.9	88.2	
	Richer	136	71.0	31.5	66.4	48.0	90.4	
	Richest	151	65.5	28.2	61.9	46.2	79.3	
Region ^2^	Northeast	101	62.0	26.7	57.1	41.9	73.8	0.001
	Southwest	142	77.2	33.6	73.4	54.4	99.6	
	Southeast	235	69.8	30.0	66.2	47.6	86.7	
	North	102	64.8	35.9	57.8	36.9	85.7	
	West	145	71.0	28.7	67.0	51.6	84.4	

^1^ Nonparametric (distribution-free) tests were performed since the Kolmogorov-Smirnov test indicated departure of vitamin D concentration data from normality (*p* = 0.000). Mann-Whitney Test was applied on two independent samples form living areas. Kruskal-Wallis test was applied on the independent samples with respect to wealth Group and region; ^2^ region: Northeast (Kratie, Mondol Kiri, Ratanak kiri, Stung Treng); Southwest (Kampong Speu, Kampot, Koh Kong, Sihanouk, Takeo); Southeast (Tboung Khmum, Svay Rieng, Prey Veng, Phnom Penh, Kandal, Kampong Chhnnang, Kampong Cham); North (Banteay Mean Chey, Preah Vihar, Ottar Mean Chey); and West (Siem Reap, Pailin, Pursat, Battambang, Kampong Thom).

**Table 2:** Comparison of relationships between the vitamin D status and living area, region, and wealth grouping among mothers.

Comparisons: Women (2)		*n*	Vitamin D Concentration				*p*-Value *
			<25 nmol/L	25–49.9 nmol/L	50–74.9 nmol/L	≥75 nmol/L	
Total		725	30	180	258	257	-
			4.1%	24.9%	35.6%	35.4%	
Living Area	Urban	148	3	47	50	48	0.100
			2.0%	31.8%	33.8%	32.4%	
	Rural	577	27	133	208	209	
			4.7%	23.1%	36.0%	36.2%	
Wealth quintile	Poorest	155	9	35	49	62	0.698
			5.8%	22.6%	31.6%	40.0%	
	Poorer	152	5	33	59	55	
			3.3%	21.7%	38.8%	36.2%	
	Middle	131	5	35	50	41	
			3.8%	26.7%	38.2%	31.3%	
	Richer	136	6	32	45	53	
			4.4%	23.5%	33.1%	39.0%	
	Richest	151	5	45	55	46	
			3.3%	29.8%	36.4%	30.5%	
Region	North East	101	3	35	40	23	0.000
			3.0%	34.7%	39.6%	22.8%	
	South West	142	7	23	42	70	
			4.9%	16.2%	29.6%	49.3%	
	South East	235	6	63	83	83	
			2.6%	26.8%	35.3%	35.3%	
	North	102	10	30	30	32	
			9.8%	29.4%	29.4%	31.4%	
	West	145	4	29	63	49	
			2.8%	20.0%	43.4%	33.8%	

Note: * the chi-square test was used.

**Table 3:** Comparison of relationships between vitamin D concentration status and living area, wealth group, and age groups of the children.

Children Comparisons (1)		Vitamin D ^1^						*p*-Value
		*n*	Mean (nmol/L)	SD (nmol/L)	Median (nmol/L)	25th Percentile P_25_ (nmol/L)	75th Percentile P_75_ (nmol/L)	
Total		781	91.1	37.3	89.5	64.7	116.0	-
Living Area	Urban	164	83.3	31.8	84.4	62.0	104.4	0.001
	Rural	617	93.1	38.4	90.8	65.0	118.0	
Wealth Group	Poorest	188	93.2	37.5	90.5	65.2	119.5	0.076
	Poorer	171	95.6	37.9	95.9	65.5	119.0	
	Middle	139	91.9	35.4	89.1	66.6	116.3	
	Richer	150	84.5	38.0	81.9	58.5	107.4	
	Richest	133	88.7	37.1	88.0	63.2	112.6	
Region	Northeast	109	81.3	33.5	80.7	55.4	103.4	0.001
	Southwest	142	97.6	35.3	93.2	73.4	120.9	
	Southeast	269	88.9	38.3	89.2	59.4	115.7	
	North	109	87.5	41.9	88.3	56.5	116.8	
	West	152	98.4	34.6	94.5	73.1	120.1	
Age Group (*only 779 have data on age*)	6–11 months	41	120.2	35.1	119.8	103.5	142.2	0.000
	12–23 months	115	103.3	41.7	104.1	72.2	131.9	
	24–59 months	495	87.9	35.4	87.2	61.4	111.4	
	60+ months	128	82.8	34.8	80.9	58.2	104.9	

^1^ Parametric tests were performed since the no evidence of departure from normality was detected by the Kolmogorov-Smirnov test (*p* = 0.669). Unequal variance *t*-test was applied on two independent samples form living areas. One-way ANOVA was applied on the independent samples with respect to wealth quintile, region and age group; ^2^ region: Northeast (Kratie, Mondol Kiri, Ratanak kiri, Stung Treng); Southwest (Kampong Speu, Kampot, Koh Kong, Sihanouk, Takeo); Southeast (Tboung Khmum, Svay Rieng, Prey Veng, Phnom Penh, Kandal, Kampong Chhnnang, Kampong Cham); North (Banteay Mean Chey, Preah Vihar, Ottar Mean Chey); and West (Siem Reap, Pailin, Pursat, Battambang, Kampong Thom).

**Table 4:** Comparative relationships between vitamin D status, living area, and wealth grouping among children.

		Total	Vitamin D Concentration				*p*-Value *
			Less Than 25 nmol/L	25–49.9 nmol/L	50–74.9 nmol/L	≥75 nmol/L	
Total		781	23	82	167	509	-
			2.9%	10.5%	21.4%	65.2%	
Living Area	Urban	164	6	19	43	96	0.234
			3.7%	11.6%	26.2%	58.5%	
	Rural	617	17	63	124	413	
			2.8%	10.2%	20.1%	66.9%	
Wealth Group	Poorest	188	4	16	46	122	0.332
			2.1%	8.5%	24.5%	64.9%	
	Poorer	171	4	17	31	119	
			2.3%	9.9%	18.1%	69.6%	
	Middle	139	2	14	29	94	
			1.4%	10.1%	20.9%	67.6%	
	Richer	150	6	21	38	85	
			4.0%	14.0%	25.3%	56.7%	
	Richest	133	7	14	23	89	
			5.3%	10.5%	17.3%	66.9%	
Region	North East	109	2	17	28	62	0.001
			1.8%	15.6%	25.7%	56.9%	
	South West	142	1	11	25	105	
			.7%	7.7%	17.6%	73.9%	
	South East	269	9	36	57	167	
			3.3%	13.4%	21.2%	62.1%	
	North	109	8	14	24	63	
			7.3%	12.8%	22.0%	57.8%	
	West	152	3	4	33	112	
			2.0%	2.6%	21.7%	73.7%	
Age group **	6–11 months	41	0	3	1	37	0.002
			0.0%	7.3%	2.4%	90.2%	
	12–23 months	115	3	10	16	86	
			2.6%	8.7%	13.9%	74.8%	
	24–59 months	495	13	51	121	310	
			2.6%	10.3%	24.4%	62.6%	
	60+ months	128	7	18	29	74	
			5.5%	14.1%	22.7%	57.8%	

(*) The Chi-Square test was used. (**) Only 779 had age category data.

## References

[1] Mithal A., Wahl D.A., Bonjour J.P., Burckhardt P., Dawson-Hughes B., Eisman J.A., Fuleihan G.E., Josse R.G., Lips P., Morales-Torres J. (2009). Global vitamin D status and determinants of hypovitaminosis D. Osteoporos. Int..

[2] Fitzpatrick T.B. (1988). The validity and practicality of sun-reactive skin types I through VI. Arch. Dermatol..

[3] Holick M.F. (2010). The Vitamin D Solution.

[4] Le Nguyen B.K., le Thi H., Do V.A.N., Thuy N.T., Huu C.N., Do T.T., Deurenberg P., Khouw I. (2013). Double burden of undernutrition and overnutrition in Vietnam in 2011: Results of the SEANUTS study in 0.5-11-year-old children. Br. J. Nutr..

[5] Rojroongwasinkul N., Kijboonchoo K., Wimonpeerapattana W., Purttiponthanee S., Yamborisut U., Boonpraderm A., Kunapan P., Thasanasuwan W., Khouw I. (2013). SEANUTS: The nutritional status and dietary intakes of 0.5–12-year-old Thai children. Br. J. Nutr..

[6] Laillou A., Wieringa F., Tran T.N., Van P.T., Le B.M., Fortin S., Le T.H., Pfanner R.M., Berger J. (2013). Hypovitaminosis D and mild hypocalcaemia are highly prevalent among young Vietnamese children and women and related to low dietary intake. PLoS ONE.

[7] Sandjaja S.B.B., Harahap H., Ernawati F., Soekatri M., Widodo Y., Sumedi E., Rustan E., Sofia G., Syarief S.N. (2013). Food consumption and nutritional and biochemical status of 0.5–12-year-old Indonesian children: the SEANUTS study. Br. J. Nutr..

[8] Poh B.K., Ng K.B., Haslinda M.D.S., Shanita S.N., Wong J.E., Budin S.B., Ruzita A.T., Ng L.O., Khouw I., Norimah A.K. (2013). Nutritional status and dietary intakes of children aged 6 months to 12 years: findings of the Nutrition Survey of Malaysian Children (SEANUTS Malaysia). Br. J. Nutr..

[9] Hien V.T., Lam N.T., Skeaff C.M., Todd J., McLean J.M., Green T.J. (2012). Vitamin D status of pregnant and non-pregnant women of reproductive age living in Hanoi City and the Hai Duong province of Vietnam. Mater. Child Nutr..

[10] Khor G.L., Chee W.S., Shariff Z.M., Poh B.K., Arumugam M., Rahman J.A., Theobald H.F. (2011). High prevalence of vitamin D insufficiency and its association with BMI-for-age among primary school children in Kuala Lumpur, Malaysia. BMC Public Health.

[11] Hawkins R.C. (2009). 25-OH vitamin D3 concentrations in Chinese, Malays, and Indians. Clin. Chem..

[12] Green T.J., Skeaff C.M., Rockell J.E., Venn B.J., Lambert A., Todd J., Khor J.L., Loh S.P., Muslimatun S., Agustina R. (2008). Vitamin D status and its association with parathyroid hormone concentrations in women of child-bearing age living in Jakarta and Kuala Lumpur. Eur. J. Clin. Nutr..

[13] Rahman S.A., Chee W.S., Yassin Z., Chan S.P. (2004). Vitamin D status among postmenopausal Malaysian women. Asia Pac. J. Clin. Nutr..

[14] Van der Rhee H., Coebergh C.J., de Vries E. (2009). Sunlight, vitamin D and the prevention of cancer: A systematic review of epidemiological studies. Eur. J. Cancer Prev..

[15] Garland C.F., Garland F.C., Gorham E.D., Lipkin M., Newmark H., Mohr S.B., Holick H.F. (2006). The role of vitamin D in cancer prevention. Am. J. Public Health.

[16] Choi Y.J., Kim Y.H., Cho C.H., Kim S.H., Lee J.E. (2015). Circulating levels of vitamin D and colorectal adenoma: A case-control study and a meta-analysis. World J. Gastroenterol..

[17] Lee J.E. (2011). Circulating levels of vitamin D, vitamin D receptor polymorphisms, and colorectal adenoma: A meta-analysis. Nutr. Res. Pract..

[18] Azeem S., Gillani S.W., Siddiqui A., Jandrajupalli S.B., Poh V., Sulaiman S.A.S. (2015). Diet and colorectal cancer risk in Asia—A systematic review. Asian Pac. J. Cancer Prev..

[19] Hossein-nezhad A., Holick M.F. (2013). Vitamin D for health: A global perspective. Mayo Clin. Proc..

[20] Wimalawansa S.J. (2012). Vitamin D: Everything You Need to Know.

[21] Pilz S., Tomaschitz A., Marz W., Drechsler C., Ritz E., Zittermann A., Cavalier E., Pieber T.R., Lappe J.M., Grant W.B. (2011). Vitamin D, cardiovascular disease and mortality. Clin. Endocrinol. (Oxf.).

[22] Hewison M. (2012). Vitamin D and immune function: An overview. Proc. Nutr. Soc..

[23] Kroner J.C., Sommer A., Fabri M. (2015). Vitamin D every day to keep the infection away?. Nutrients.

[24] Wimalawansa S.J. (2012). Vitamin D: An essential component for skeletal health. Ann. N. Y. Acad. Sci..

[25] Weaver C., Lewis R., Laing E. (2011). Adolescence and Acquisition of Peak Bone Mass. Vitamin D.

[26] Nguyen H.T., von Schoultz B., Pham D.M., Nguyen D.B., Le Q.H., Nguyen D.V., Hirschberg A.L., Nguyen T.V. (2009). Peak bone mineral density in Vietnamese women. Arch. Osteoporos..

[27] Gafni R.I., Baron J. (2007). Childhood bone mass acquisition and peak bone mass may not be important determinants of bone mass in late adulthood. Pediatrics.

[28] Mithal A., Dhingra V., Lau E., Stenmark J. (2009). The Asian Audit: Epidemiology, Costs and Burden of Osteoporosis in Asia.

[29] Osborne D.L., Weaver C.M., McCabe L.D., McCabe G.P., Novotny R., van Loan M.D., Going S., Matkovic V., Boushey C.J., Savaiano D.A. (2012). Body size and pubertal development explain ethnic differences in structural geometry at the femur in Asian, Hispanic, and white early adolescent girls living in the U.S.. Bone.

[30] World Health Organisation (2003). Prevention and Management of Osteoporosis.

[31] National Institute of Statistics, Directorate General for Health, ICF International (2015). Cambodia Demographic and Health Survey 2014.

[32] Holick M.F., Binkley N.C., Bischoff-Ferrari H.A., Gordon C.M., Hanley D.A., Heaney R.P., Murad M.H., Weaver C.M. (2011). Evaluation, treatment, and prevention of vitamin D deficiency: An Endocrine Society clinical practice guideline. J. Clin. Endocrinol. Metab..

[33] Wimalawansa S.J. (2012). Vitamin D in the new millennium. Curr. Osteoporos. Rep..

[34] Dietary Reference Intakes for Calcium and Vitamin D. http://tinyurl.com/hqd6p3j.

[35] Bischoff-Ferrari H.A., Giovannucci E., Willett W.C., Dietrich T., Dawson-Hughes B. (2006). Estimation of optimal serum concentrations of 25-hydroxyvitamin D for multiple health outcomes. Am. J. Clin. Nutr..

[36] Holick M.F. (2006). High prevalence of vitamin D inadequacy and implications for health. Mayo Clin. Proc..

[37] Rutstein S.O. The DHS Wealth Index: Approaches for Rural and Urban Areas. http://tinyurl.com/jdzsrt7.

[38] Powe C.E., Evans M.K., Wenger J., Zonderman A.B., Berg A.H., Nalls M., Tamez H., Zhang D., Bhan I., Karumanchi S.A. (2013). Vitamin D–binding protein and vitamin D status of black Americans and white Americans. N. Engl. J. Med..

[39] Thongthai P., Chailurkit L., Chanprasertyothin S., Nimitphong H., Sritara P., Aekplakorn W., Ongphiphadhanakul B. (2015). Vitamin D binding protein gene polymorphism as a risk factor for vitamin D deficiency in Thais. Endocr. Pract..

[40] Wu L., Martin B.R., Braun M.M., Wastney M.E., McCabe G.P., McCabe L.D., DiMeglio L.A., Peacock M., Weaver C.M. (2010). Calcium requirements and metabolism in Chinese-American boys and girls. J. Bone Miner Res..

[41] Holick M.F. (2014). Sunlight, ultraviolet radiation, vitamin D and skin cancer: How much sunlight do we need?. Adv. Exp. Med. Biol..

[42] Egan K.M. (2009). Vitamin D and melanoma. Ann. Epidemiol..

[43] Gillie O. (2012). The Scots’ Paradox: Can sun exposure, or lack of it, explain major paradoxes in epidemiology?. Anticancer Res..

[44] Lee H., Chay Y.W., Tang M.B.Y., Chio M.T.W., Tan S.H. (2012). Melanoma: Differences between Asian and Caucasian patients. Ann. Acad. Med. Singapore.

[45] Baggerly C.A., Cuomo R.E., French C.B., Garland C.F., Gorham E.D., Grant W.B., Heaney R.P., Holick M.F., Hollis B.W., McDonnell S.L. (2015). Sunlight and vitamin D: Necessary for public health. J. Am. Coll. Nutr..

[46] Grant W.B., Wimalawansa S.J., Holick M.F. (2015). Vitamin D supplements and reasonable solar UVB should be recommended to prevent escalating incidence of chronic diseases. Br. Med. J..

[47] Nimitphong H., Holick M.F. (2013). Vitamin D status and sun exposure in Southeast Asia. Dermatoendocrinology.

[48] Ross A.C., Manson J.E., Abrams S.A., Aloia J.F., Brannon P.M., Clinton S.K., Durazo-Arvizu R.A., Gallagher J.C., Gallo R.L., Jones G. (2011). The 2011 report on dietary reference intakes for calcium and vitamin D from the Institute of Medicine: What clinicians need to know. J. Clin. Endocrinol. Metab..

[49] Holik M.F. (2007). Vitamin D deficiency. N. Engl. J. Med..

[50] Chen T.C., Chimeh F., Lu Z., Mathieu J., Person K.S., Zhang A., Kohn N., Martinello S., Berkowitz R., Holick M.F. (2007). Factors that influence the cutaneous synthesis and dietary sources of vitamin D. Arch. Biochem. Biophys..

[51] Mau J.L., Chen P.R., Yang J.H. (1998). Ultraviolet irradiation increased vitamin D_2_ content in edible mushrooms. J. Agric. Food Chem..

[52] Wimalawansa S.J. (2013). Food fortification programs to alleviate micronutrient deficiencies. J. Food Process Technol..

[53] Yang Z., Laillou A., Smith G., Schofield D., Moench-Pfanner R. (2013). A review of vitamin D fortification: Implications for nutrition programming in Southeast Asia. Food Nutr. Bull..

[54] Farrell C.J., Herrmann M. (2013). Determination of vitamin D and its metabolites. Best Pract. Res. Clin. Endocrinol. Metab..

[55] Wielders J.P., Carter G.F., Eberl H., Morris G., Roth H.J., Vogl C. (2015). Automated competitive protein-binding assay for total 25-OH vitamin D, multicenter evaluation and practical performance. J. Clin. Lab. Anal..

